# Preservation of Bodies for Anatomical Purposes

**Published:** 1860-06

**Authors:** 


*Preservation of Bodies for Anatomical Purposes.*—Prof. Budge has found that bodies may be admirably preserved for a long period of time, whether for anatomical purposes, or for courses of operative surgery, by injecting into the carotid a preservative fluid composed of pyroligneous acid and sulphate of zinc, of each from eight to twelve drachms to seven pounds of water. Bodies thus injected have kept during eight weeks of intense summer heat, without giving rise to any putrefactive smell, the muscles retaining their red color, and though a little softened, admitting of good dissection. The injection does not prevent the subsequent injection of colored matters; and the knives used in dissection scarcely suffer at all.—*Virchow's Archiv.*

